# Measuring healthcare quality in police custody: the development of quality indicators through expert consultations in the Netherlands

**DOI:** 10.1186/s40352-026-00415-2

**Published:** 2026-08-05

**Authors:** Nienke D. Zinger, Dionne S. Kringos, Robert A. Verheij, Isabelle Bos, Vina N. Slev

**Affiliations:** 1 Netherlands Institute for Health Services Research, Nivel, Utrecht, Netherlands; 2 Public and Occupational Health, Amsterdam UMC Location University of Amsterdam, Amsterdam, Netherlands; 3 Quality of Care, Global Health, Amsterdam Public Health, Amsterdam, Netherlands; 4 Tilburg School of Social and Behavioral Sciences, Tilburg University, Tranzo, Tilburg, Netherlands; 5 Public Health Service, Department of Forensic Medicine, Amsterdam, Netherlands

**Keywords:** Quality of care, Quality indicators, Police custody

## Abstract

**Background:**

Improving the quality of care for people detained in police custody requires careful measurement and monitoring. However, a standardized and endorsed set of quality indicators for healthcare in police custody settings is lacking. This study aimed to develop a core set of quality indicators to monitor and evaluate police custody healthcare in the Netherlands.

**Methods:**

A four-phase modified Delphi process was conducted between February 2024 and September 2025: (1) identification of candidate quality indicators ethrough expert brainstorming; (2) rating of indicators on relevance and measurability in a self-administered questionnaire; (3) an in-person group consultation meeting to refine and prioritize the indicators; and (4) content validation of the core set by an advisory committee. A multidisciplinary pool of professionals from healthcare, police, justice, and adjacent sectors participated throughout.

**Results:**

Brainstorming sessions with 14 experts yielded 185 candidate indicators, which were rated by 19 experts in the questionnaire. The group consultation meeting with 15 participants refined the list, and feedback from 8 advisory committee members informed the final selection. The resulting core set comprised 15 structure, 9–12 process (7 fixed and 2–5 variable), and 17 outcome indicators, covering all key domains of police custody healthcare quality.

**Conclusions:**

The first evidence-based set of indicators provides a foundation for the systematic assessment and continuous learning in police custody healthcare. Routine healthcare data may serve as a valuable measurement source, though their usefulness depends on data quality, accessibility, and system readiness.

**Supplementary Information:**

The online version contains supplementary material available at 10.1186/s40352-026-00415-2.

## Background

Incarcerated persons have the right to healthcare services of the same quality as those available outside detention settings (Abbing, 2013). Despite the brief duration of police custody, detained individuals may face serious health risks if essential care is delayed or withheld. Police custody detainees often experience poor physical and mental health conditions and may be at elevated risk of acute drug and alcohol intoxication or withdrawal (Zinger et al., 2026; McKinnon et al., 2016; Rekrut-Lapa & Lapa, 2014; Sondhi & Williams, 2018). One of the distinctive features of healthcare in police custody is that consultations must be requested through custody officers, a procedural step that can delay timely access to care (Pont et al., 2018). Ensuring good quality healthcare is therefore essential to prevent health deterioration and to safeguard compliance with healthcare standards (Pont et al., 2018).

Performance measurement is a cornerstone of quality improvement and accountability in healthcare (WHO, 2008; Foley & Vale, 2023). Routinely monitoring through quality indicators provides essential information to identify priority areas, guide corrective actions, and track progress over time (Forster & van Walraven, 2012; Stelfox & Straus, 2013b). Data collected from practice can be analyzed to support learning in health systems by providing feedback to healthcare providers; driving improvement in patient outcomes, resource allocation, and staff performance (Enticott et al., 2021; WHO, 2008; Foley & Vale, 2023; Foley & Vale, 2017). In longer-term prison settings, structured performance assessment has been supported by initiatives such as the guide and toolkit co-developed by the International Committee of the Red Cross (ICRC, 2018), and the Health In Prisons European Database (HIPED), which collects data guided by the World Health Organization (WHO) Prison Health Framework (Alves da Costa et al., 2022).

Despite these developments, important gaps in prison health quality measurement remain. A previous study has noted inconsistent rigor in indicator development, limited involvement of detained persons, and a lack of structure and outcome indicators. Moreover, it showed that scientific literature on quality indicator development for primary healthcare in prison settings appears largely limited to the United States (Bellass et al., 2022). The healthcare needs of police custody detainees differ from those in prisons, because of the short-term nature of detention and the requirement to provide healthcare only when delaying treatment would harm the detainee’s health (Thoonen, 2017).

In the Netherlands, no system currently exists for structured measurement, monitoring, or evaluation of healthcare quality in police custody; a gap that was recently recognized in the national research agenda of the Dutch Forensic Medical Society. Similar calls for national standards and datasets to monitor and evaluate police custody healthcare have been made in the United Kingdom (Kennedy et al., 2023; Payne-James, 2017). However, a recent review of the international literature found no quality indicators specifically developed for short-term police custody settings (Zinger et al., 2026).

To address this gap, this study aims to systematically develop a core set of quality indicators for healthcare in police custody through expert consultation, building on a previously developed conceptual framework that identifies key aspects for monitoring and improving quality of care (Zinger et al., 2026).

## Methods

A modified Delphi approach was applied to develop a core set of quality indicators, a widely used technique for structured and transparent indicator development (Boulkedid et al., 2011; Fitch et al., 2001). The domains and subdomains of a previously developed conceptual framework (Zinger et al., 2026) guided the process (Schang et al., 2021). Between February 2024 and September 2025, four phases were completed: (1) identification of candidate indicators; (2) a self-administered questionnaire; (3) a group consultation meeting; and (4) selection and content validation of the final core set. A multidisciplinary expert panel participated in the first three phases, and the advisory committee was involved in phases three and four.

### Expert panel recruitment

Professionals involved in providing or supporting healthcare for police custody detainees were invited to join the expert panel through outreach to the advisory committee, professional organizations, snowball sampling, and the research team’s networks. Participants included healthcare providers, police officers, persons with lived experience of detention in police custody, and professionals affiliated with custodial institutions, mental health crisis teams, and pharmacy services.

A subset of the expert panel was asked to participate in the individual brainstorming sessions (phase 1). All panel members were informed that participation comprised two rounds: a self-administered questionnaire (phase 2), and an in-person group consultation meeting (phase 3). They were encouraged, but not required, to join both rounds to accommodate potential constraints such as irregular working hours and staff shortages.

### Advisory committee

The advisory committee included management representatives from police custody healthcare organizations, the police, the Dutch Public Prosecution Service, the Dutch Custodial Institutions Agency, and a detainee advocacy group. Members were invited to join the group consultation (phase 3) and to validate the final core set (phase 4), ensuring broader system-level perspectives.

### Phase 1: Identification of candidate quality indicators

Candidate quality indicators were derived from recommendations identified in a previous scoping review (Zinger et al., 2026), the authors’ expertise in health systems performance assessment and police custody healthcare, and individual brainstorming sessions with a subset of expert panel members. Indicators were organized according to the conceptual framework, encompassing Donabedian’s structure, process, and outcome domains (Donabedian, 1966). Sessions (30–120 min) focused on participants’ areas of expertise, and new suggestions were incorporated iteratively until data saturation was reached.

### Phase 2: Self-administered questionnaire

All candidate indicators were included in a self-administered electronic questionnaire. Expert panel participants rated each indicator on relevance (importance for assessing quality of care) and measurability (feasibility of measurement using existing or future data sources) on a 5-point Likert scale. Free-text fields allowed comments and suggestions, and participants could select ‘not my expertise’ for the indicators outside their scope of knowledge.

The questionnaire was distributed electronically via email to 28 panel members, accompanied by a manual, and an instructional video. Question-and-answer sessions were offered via Microsoft Teams. The questionnaire was developed and administered using Microsoft Excel, leveraging built-in features such as data validation and conditional formatting to enhance usability. Information collected from respondents at the start of the questionnaire included their profession and years of experience.

Mean ratings and standard deviations (sd) for the relevance and measurability scoring criteria were calculated for each indicator. Consensus was predefined: quality indicators for which at least 80% of the relevance ratings were in the upper categories (4 or 5; blanks and ‘not my expertise’ excluded) were flagged to denote their strong potential for inclusion in the core set. Similarly, when at least 80% of the indicator’s relevance ratings fell within in the lower categories (1 or 2), the indicator was flagged as having low potential for selection in the core set. The indicators that did not meet the cut-off criteria were prioritized for emphasis in the group consultation meeting.

Measurability scores were recorded for reference and played a larger role in defining the core set (phase 4). The questionnaire responses were imported into RStudio for preparation, and descriptive statistics were generated using STATA version 16. Preceding the group consultation meeting (phase 3), participants received personalized reports of the questionnaire results, detailing the group mean and standard deviation for each indicator alongside their individual ratings.

### Phase 3: Group consultation meeting

The in-person group consultation meeting (4.5 h) aimed to facilitate structured discussion and refinement of the long list of indicators. Chaired by an independent facilitator with expertise in health system performance assessment, the meeting followed the conceptual framework domains to ensure comprehensive coverage. Indicators were discussed in groups according to their interdependencies, with the sequence moving from outcome to process to structure indicators to encourage engagement. Two authors (DK, VS) took notes, which were consolidated, categorized by domain, and merged with the questionnaire results to form the final long list.

### Phase 4: Selection and validation of core set of indicators

The authors synthesized findings from the previous phases to propose a core set of indicators. Each indicator was reviewed against five criteria to ensure domain coverage of the conceptual framework, and a balanced mix of structure, process, and outcome indicators. The criteria were: (1) relevance (priority was given to indicators that received high scores in the questionnaire); (2) measurability (preference was given to indicators that could be measured using existing data systems, such as electronic health records or other registration systems); (3) quality impact (emphasis was placed on indicators whose outcome could inform or trigger quality improvement efforts); (4) stakeholder relevance (indicators with importance to diverse stakeholder groups were preferred); and (5) implementation readiness (priority was given to indicators for which the necessary infrastructure is already in place or can feasibly be implemented, allowing for short-term implementation).

The proposed core set, including justifications for inclusion, were circulated to advisory committee members for review and feedback. Members indicated agreement or disagreement with inclusion, completeness, and suggested additions or modifications where relevant. Feedback was reviewed and integrated, and a follow-up meeting addressed remaining issues. Final agreement by all participants established the validated core set.

## Results

**Fig. 1:**
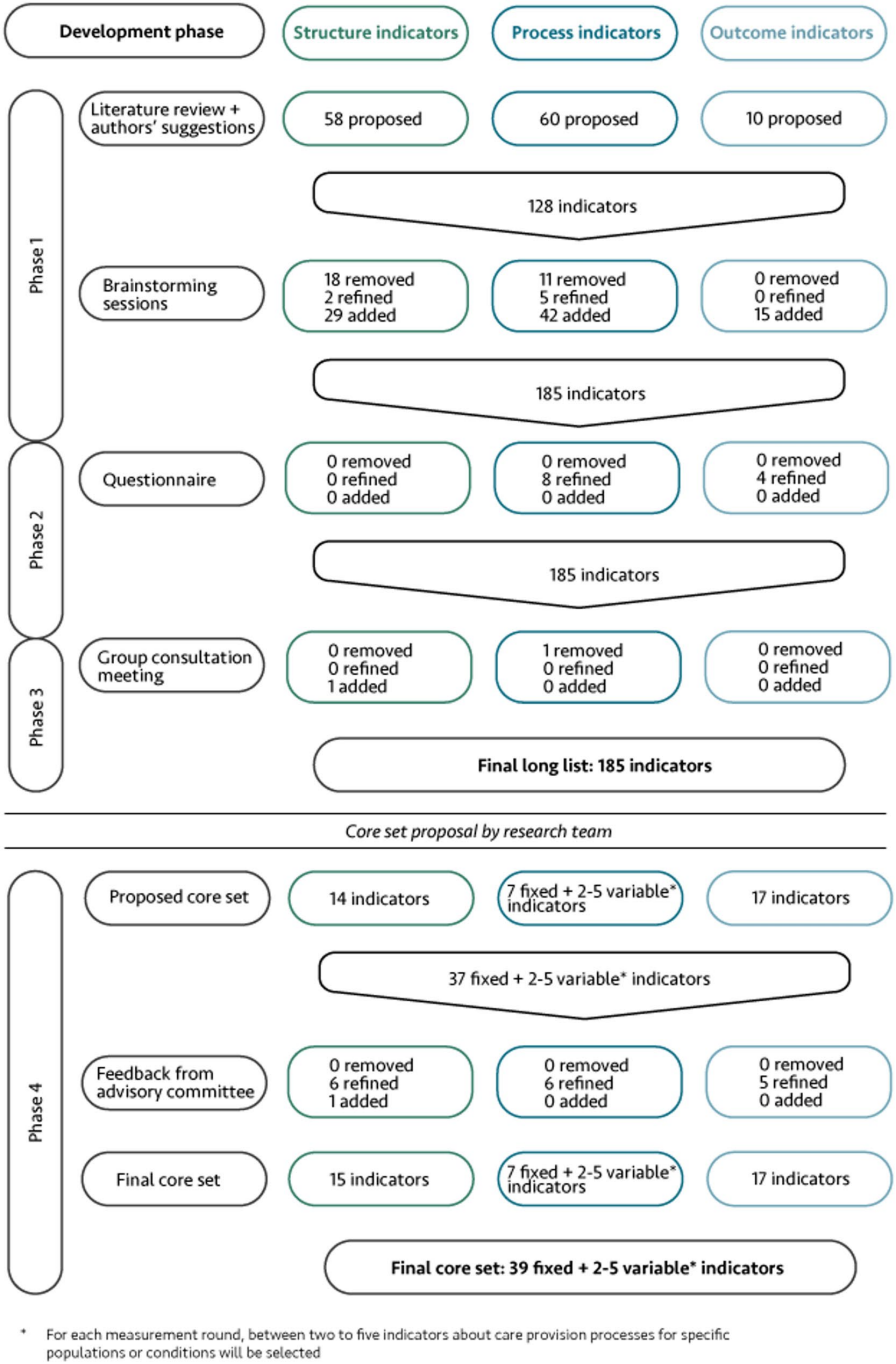
Overview of the number of quality indicators in the selection process

Table 1 presents the areas of expertise of the participants involved in the indicator development and selection process. Fig. 1 shows the indicator selection process.

**Table 1:** Overview of participants in each phase of the quality indicator development process

Phase 1: Individual brainstorming sessions (*n* = 14) Expert panel	
• Physicians providing healthcare in police custody (*n* = 4) *Forensic physicians*,* general practitioners* • Forensic nurses providing healthcare in police custody (*n* = 2) • Police (*n* = 3) *Police custody officers*,* operational expert*,* assistant public prosecutor* • People with lived experience of police detention (*n* = 2) • Scientist specialized in forensic medicine (*n* = 1) • Psychiatrist providing mental health crisis services (*n* = 1) • Pharmacist (*n* = 1)	

Phase 2: Self-administered questionnaire (*n* = 19) Expert panel	
• Physicians providing healthcare in police custody (*n* = 6) *Forensic physicians*,* general practitioners* • Forensic nurses providing healthcare in police custody (*n* = 5) • Police (*n* = 3) *Police custody officers*,* operational expert*,* assistant public prosecutor* • Person with lived experience of police detention (*n* = 1) • Educators of forensic medicine or forensic nursing (*n* = 2) • Physician Dutch Street Doctors Group (*n* = 1) • Policy officer of the Public Prosecution Service (*n* = 1) *Medical Affairs Expertise Center*	

Phase 3: Group consultation meeting (*n* = 15) Expert panel and advisory committee	
• Physicians providing healthcare in police custody (*n* = 4) *Forensic physicians*,* general practitioners* • Forensic nurse providing healthcare in police custody (*n* = 1) • Healthcare organization managers and care coordinators (*n* = 3) • Police (*n* = 1) *Police custody officer and operational expert* • Advocacy for detainees (*n* = 1) *Dutch independent advocacy group for (former) detainees and their families* • Educator of forensic nursing (*n* = 1) • Dutch Custodial Institutions Agency (*n* = 1) *Medical advisor and forensic physician* • Judicial nurses providing healthcare in custodial facilities (*n* = 2) • Pharmacist (*n* = 1)	

Phase 4: Content validation of indicator core set (*n* = 8) Advisory committee	
• Healthcare organization managers and care coordinators (*n* = 4) • Dutch Forensic Medical Society representatives (*n* = 1) • Police (*n* = 1) *Portfolio of detainee care* • Policy officer of the Public Prosecution Service (*n* = 1) *Medical Affairs Expertise Center* • Dutch Custodial Institutions Agency (*n* = 1) *Medical advisor and forensic physician*	

### Phase 1: Identification of candidate quality indicators

The prior literature review and the author’s input yielded 128 initial indicators. Brainstorming sessions with 14 experts (Table 1) resulted in the removal of 29 indicators deemed too narrowly focused, context-specific, or dependent on patient-level information. Eighty-six new indicators were added, resulting in a list of 185 indicators, about structures (*n* = 69), processes (*n* = 91), and outcomes (*n* = 25) to be included for the second phase (Fig. 1).

### Phase 2: Self-administered questionnaire

Nineteen experts returned the self-administered questionnaire (response rate: 68%; Table 1), most (84%) with over five years’ experience. Non-participation was mainly due to time constraints (*n* = 4), or limited subject-matter relevance (*n* = 2). No participants joined the optional question-and-answer sessions, though one participant received clarification by video call.

#### Relevance ratings

Each indicator’s relevance was rated by a median of 14 experts, with the number of responses ranging from 5 to 18. Mean scores ranged from 2.8 to 4.9, with a median of 4. A total of 47% of the indicators were rated 3 or higher. A third of the indicators (34%) were scored above the predefined threshold. Among the structure indicators, 64% (*n* = 44) scored above the threshold, specifically within the domains of quality assurance (25 of 35 indicators), and infrastructure and (medial) resources (6 of 9 indicators). The threshold was also met for 12% (*n* = 11) of the process indicators, and 28% (*n* = 7) of the outcome indicators.

None of the indicators scored below the threshold for probable exclusion in the core set (Fig. 1). Relatively low scores were given to indicators concerning, among others, the healthcare financing structure, professionals’ satisfaction with the registration burden, and translator involvement.

#### Measurability ratings

A median of 11 experts rated each indicator’s measurability, with 4 to 14 responses per indicator. Mean measurability scores ranged from 2.2 to 4.7, with a median of 4.1. Just over half of the indicators (51%, *n* = 95) were rated a 3 or higher, and 92 (50%) scored above the threshold. Within the structure indicators, 81% (*n* = 69) exceeded the threshold, including those addressing the existence of protocols and the training of police and healthcare professionals. Process indicators meeting the threshold (35%, *n* = 32) included those evaluating whether particular procedures are carried out.

### Phase 3: Group consultation meeting

Fifteen participants (60% of invitees) attended the in-person group consultation meeting, including six questionnaire participants (phase 2), five additional experts, and four advisory committee members (Table 1). One indicator was removed and one was added (Fig. 1). Discussions emphasized key quality domains across structure, process, and outcome indicators.

#### Structure indicators

Three priority areas related to structures emerged. The first concerned the training of custody officers, considered essential for identifying urgent medical needs while respecting that triage and medical assessments remain the responsibility of the healthcare providers. The second related to monitoring healthcare staffing capacity to safeguard service quality. The third focused on strengthening quality assurance structures, particularly through regular multidisciplinary peer-review meetings between healthcare staff and custody officers.

#### Process indicators

Discussions on process indicators centered on timely access to healthcare, information sharing, and care coordination. Timeliness, identification of care needs, and triage were deemed critical but difficult to measure due to fragmented or paper-based registration systems and retrospective recording. Measuring timely access to medication was also seen as challenging because delays often stem from context-specific or external factors (e.g., timeliness is contingent upon the urgency of the medication’s need, and pharmacy availability).

Participants highlighted structural barriers to information sharing between police, healthcare providers, and custodial facilities, noting that limited transfer of medical information (e.g., from hospitals to police custody, or from police custody to custodial facilities) hinders continuity of care and quality improvement. Indicators capturing the number and type of registered health complaints and care activities were seen as useful for mapping regional trends and supporting reflection. Accurate record-keeping was emphasized as vital, given that some detainees cannot clearly articulate their needs and may be treated by multiple providers during detention.

#### Outcome indicators

Mortality and complication outcome indicators were regarded as essential for assessing adverse events. Participants supported developing a national structure for confidential, inter-regional case discussions to support learning, but noted the absence of such feedback systems. Satisfaction indicators for detainees, healthcare professionals, and police were also considered informative, provided that a standardized complaints mechanism is established. Participants recognized challenges in interpreting detainee satisfaction, as perceptions may conflate experiences of detention with healthcare quality, but agreed that the measure remains valuable.

### Phase 4: Selection and content validation of the core set of quality indicators

The first three phases yielded a final long list of 185 quality indicators (Supplementary Material 1). From this, 37 fixed plus 2 to 5 variable indicators were selected to constitute the proposed core set. The variable indicators allow periodic focus on specific populations or conditions (e.g., mental health problems, severe intoxications, minors). Eight of ten advisory committee members provided written feedback on the proposed core set of indicators, and nine attended the validation meeting. The final core set of indicators is presented in Table 2 and contains 15 indicators about structures, 7 fixed and 2 to 5 variable indicators about processes, and 17 outcome indicators. No quality indicators were selected for the funding domain, a decision endorsed by all committee members.

**Table 2:** Core set of quality indicators for healthcare in police custody, organized by domain

Domain	Indicator	Reporting format
STRUCTURE INDICATORS		
Legal framework	Do the healthcare provider organizations have access to the National Exchange Point? *(The National Exchange Point allows healthcare providers to consult medical information about their patients across organizations)*	Narrative description
Scope and nature of the care demand	Is information on diagnoses and treatments/procedures performed by healthcare providers systematically recorded? At least: - Distinction between somatic and mental health complaints / disorders; - Structured classification of health complaints, symptoms and disorders (e.g., use of International Classification of Primary Care); - Distinction between types of procedures.	Narrative description
(Healthcare) staff	Number of healthcare providers who completed education or training specifically focused on healthcare provision in police custody, as a percentage of the total number of police custody healthcare providers	Descriptive statistics and a narrative description of the education and training content
(Healthcare) staff	Do the police have an organization-wide policy on training and education related to knowledge of specific populations, their health needs, associated health risks, and conduct toward them?	Narrative description
(Healthcare) staff	Do the healthcare provider organizations have an organization-wide policy on training and education related to knowledge of specific populations, their health needs, associated health risks, and conduct toward them?	Narrative description
Quality assurance	Do the police have a standardized questionnaire about detainees’ medical care needs that the police custody officers can use at intake to make an initial assessment of the health situation and health risks of detainees?	Narrative description
Quality assurance	Has the standardized questionnaire on detainees’ medical care needs, that the police can use at intake, been developed in consultation with healthcare providers?	Narrative description
Quality assurance	Are there established agreements between the police, the healthcare provider organization, and pharmacies about the delivery and/or pickup of medication, both during and outside pharmacy office hours?	Narrative description
Quality assurance	In cases where the police distribute the medication to detainees: are there established agreements between the police and the healthcare provider organization about how and when this distribution of medication should be executed?	Narrative description
Quality assurance	Are there between the police, the healthcare provider organization, and the mental health crisis service established agreements about the scope and setup of the collaboration and the involvement of each party in the care provision for individuals with confused or challenging behavior held in police custody?	Narrative description
Quality assurance	Do the healthcare provider organizations have established opportunities for peer consultation, both routinely and in acute situations?	Narrative description
Quality assurance	Do the police have a procedure in place for (former-)detainees to report complaints about the care and medical care within the police custody facility?	Narrative description
Quality assurance	Do the police organize at least once a year peer review meetings, during which attention is also paid to (medical) care provision?	Narrative description
Quality assurance	Do the healthcare provider organizations organize at least once a year peer review meetings, during which attention is paid to medical care issues that have arisen?	Narrative description
Infrastructure and (medical) resources	Is there an established structure in place that available medical equipment and materials (e.g., blood glucose monitors) are periodically maintained, verified, or calibrated?	Narrative description

PROCESS INDICATORS		
Triage and access to healthcare	Number of detainees for whom the standardized questionnaire about medical care needs has been fully completed by the police at intake, as a percentage of the total number of detainees	Descriptive statistics
Triage and access to healthcare	Number of times the action resulting from the questionnaire about the detainee’s medical care needs was recorded by the police, as a percentage of the total number of detainees for whom the questionnaire was fully completed	Descriptive statistics
Detection of care needs	Number of detainees for whom a healthcare provider was contacted, as a percentage of the total number of detainees	Descriptive statistics
Continuity of healthcare information	Number of times the health records kept by the healthcare provider contains at least detainee’s: date of birth, sex, and International Classification of Primary Care code, as a percentage of the total number of reviewed records	Descriptive statistics
Coordination of care	In case of transfer to a custodial facility (e.g., pre-trial detention facility): number of times information from the health record has been shared with the healthcare provider in the custodial facility, as a percentage of the total number of detainees who were transferred from police custody to a custodial facility	Descriptive statistics
Healthcare provision (general)	Insights into activities related to medical care: - Frequently registered International Classification of Primary Care codes - Percentage of face-to-face and telephone consultations - Type(s) of involved healthcare providers (e.g., physician, nurse) - Percentage of detainees referred to the hospital - Percentage of detainees who received a fitness for custody assessment, and description of the outcome of the assessment	Descriptive statistics
Healthcare provision (general)	In cases where medication was prescribed: number of times the medication administration instruction was registered in the health record, as a percentage of the total number of detainees for whom medication was prescribed	Descriptive statistics
Healthcare provision (common needs)	*This domain comprises quality indicators that are specific to certain care needs*,* themes*,* or population groups. In each measurement round*,* quality indicators from this domain will be rotated*,* with one or more themes being measured in each round. Themes are: drug withdrawal symptoms*,* alcohol withdrawal symptoms*,* mental health problems / individuals with confused or challenging behavior*,* suspicion of suicidal tendencies*,* intellectual disability*,* and minors (**Supplementary Material 1** indicators # 148 to 160).*	Descriptive statistics

OUTCOME INDICATORS		
Effectiveness and health	Number of deaths in police custody	Descriptive statistics, including information about region, age, (medical) characteristics
Effectiveness and health	Description of recorded deaths in police custody	Narrative description
Effectiveness and health	Assessment of the preventability of (potentially) healthcare-related deaths in police custody	Narrative description
Effectiveness and health	Number of registered healthcare-related incidents, as a percentage of the total number of detainees (incident defined as: an unintended or unexpected event that relates to the quality of care, and which has led, could have led, or might lead to harm to the client)	Descriptive statistics, including information about the region
Effectiveness and health	Description of recorded healthcare-related incidents (incident defined as: an unintended or unexpected event that relates to the quality of care, and which has led, could have led, or might lead to harm to the client)	Narrative description
Effectiveness and health	Assessment of preventability of (potentially) healthcare-related incidents	Narrative description, analysis carried out by experts
Satisfaction	Number of care-related complaints of detainees about the police, as a percentage of the total number of detainees	Descriptive statistics, including information about the region
Satisfaction	Description of care-related complaints of detainees about the police	Narrative description
Satisfaction	Number of healthcare-related complaints of detainees about the healthcare providers, as a percentage of the total number of detainees	Descriptive statistics, including information about the region
Satisfaction	Description of healthcare-related complaints of detainees about the healthcare providers, as a percentage of the total number of detainees	Narrative description
Satisfaction	Satisfaction of detainees with the conduct of the police	Descriptive statistics or narrative description
Satisfaction	Satisfaction of detainees with the conduct of the healthcare providers	Descriptive statistics or narrative description
Satisfaction	Number of times healthcare providers felt they were contacted in a timely manner, as a percentage of the total number of times the question was asked	Descriptive statistics
Satisfaction	Satisfaction of the police about the provided healthcare by the healthcare providers (other than response times)	Descriptive statistics or narrative description
Satisfaction	Satisfaction of the police about the response times of the healthcare providers	Descriptive statistics or narrative description
Satisfaction	Satisfaction of the police about the agreements for the delivery and/or pickup of medication during pharmacy office hours	Descriptive statistics or narrative description
Satisfaction	Satisfaction of the police about the agreements for the delivery and/or pickup of medication outside pharmacy office hours	Descriptive statistics or narrative description

## Discussion

This study developed a core set of 15 structure, 9–12 process (7 fixed and 2–5 variable), and 17 outcome indicators to assess fundamental aspects of quality of care for people detained in police custody in the Netherlands. To our knowledge, this is the first systematic effort to develop quality indicators for healthcare in this setting. The set was derived through a structured modified Delphi process, guided by a conceptual framework specifically developed for police custody healthcare (Zinger, et al., 2026). Together, the four phases – brainstorming, questionnaire, consultation, and validation – ensured completeness, operational clarity, and multi-stakeholder endorsement.

Indicators on structures were considered particularly relevant, contrasting with previous work by Bellass et al. (2022), who found few structural indicators when reviewing prison healthcare performance measures in the literature. Whereas Bellass et al. (2022) suggested that the contextual character of structural indicator data may be of limited interest to professionals focused on patient needs and not directly responsible for organizational preconditions, our engagement of a broad range of professionals with diverse roles could have ensured these preconditions were considered.

Police custody care involves multiple actors with distinct responsibilities and perspectives. Indicators related to transitions of detainees (i.e. from police to healthcare providers, or from police custody to pre-trial detention) or interprofessional collaboration were consistently prioritized. These highlighted the need for clear protocols and communication channels to ensure continuity of care. Quality in this context must integrate different and sometimes competing perspectives (WHO, 2008); the police emphasize rapid access to healthcare; healthcare professionals value timely notification and proper handover; custodial and community partners stress information exchange; and detainees prioritize respectful treatment. Although participants questioned whether detainees can distinguish dissatisfaction with detention from dissatisfaction with care, they agreed that detainee satisfaction remains a key dimension of quality. Similar findings have been reported in an ethnographic study among Norwegian prisoners, where participants spoke about the correctional system even when discussing healthcare (Nyvoll, 2025).

Quality of care can be assessed through various methods, including routine health care data, interviews, and questionnaires (Stelfox and Straus 2013a). For the core set of indicators, priority was given to measures obtainable from existing registry data to minimize administrative burden. The limited number of process indicators preferred by participants may partly reflect concerns about their measurability within current registration systems. To ensure comprehensive coverage of all fundamental aspects of police custody care, the set was supplemented with indicators that are currently less easily measurable, underscoring their relevance and need to strengthen the registration systems (Stelfox and Straus 2013a). A subsequent step involves evaluating the practical applicability of these indicators, particularly the suitability of routine data and potential sources of bias (Barbazza et al., 2021; Deeny & Steventon, 2015; WHO, 2008; Kötter et al., 2012; Lewis et al., 2023; Verheij et al., 2018).

When well-designed and properly implemented, quality measurement can drive accountability and improvement in health systems (WHO, 2008; Foley & Vale, 2023). To develop a data-driven learning health system within police custody care, regular measurement of the core set and systematic evaluation of the outcomes are required (Lannon et al., 2021). This enables the identification of trends, feedback to professionals and organizations, and continuous refinement of practices and policies (Foley & Vale, 2023; Lannon et al., 2021). To achieve this, roles and responsibilities for data collection, coordination, and feedback must be clearly defined (WHO, 2008). As the organizational system evolves, the core indicators may require periodic revision to remain relevant, yet such changes must be weighed against the need for stability to monitor outcomes over time (Mattke 2008; Stelfox and Straus 2013a). As the system matures and foundational structures and organizational preconditions become more established, stakeholders’ attention may gradually shift toward process-oriented indicators to evaluate whether intended practices are consistently implemented. When applying the indicator set in other countries, contextual adaptation might be required, as healthcare regulation, methods, and standards vary internationally (Heide et al., 2018; Wardrop et al., 2021). The long list of indicators provided in the supplementary material could serve as a starting point.

### Strengths and limitations

Some limitations of this study should be acknowledged. This study departs in some respects from the RAND/UCLA modified Delphi method (Fitch et al., 2001), a common feature in indicator development (Boulkedid et al., 2011). Panel composition varied between phases, which may have led to over- or underrepresentation of certain perspectives or inconsistencies in familiarity with earlier discussions. However, this flexibility allowed inclusion of groups often missing in similar work, such as individuals with lived experience in police custody (Kötter et al., 2012), and professionals from adjacent sectors such as mental health crisis services and street medicine. Future work should further explore indicators that capture collaboration between police custody care and such adjacent services, given the high prevalence of people with confused or challenging behavior in custody who may not have committed a serious offense (O’Hagan, 2018; Verheesen et al., 2021).

A notable strength of this study lies in using a setting-specific conceptual framework, to guide indicator selection, ensuring coverage across all domains of quality (WHO, 2008; Langendam et al., 2020; Schang et al., 2021). Another strength is the multidisciplinary engagement throughout all phases, with police, healthcare professionals, and system-level actors participating actively. This diversity not only enhanced the indicators’ relevance and alignment with practice but also fostered ownership among stakeholders – the foundation for future implementation and sustained use (WHO, 2008).

## Conclusions

This first evidence-based set of quality indicators provides a foundation for systematic assessment and continuous improvement of healthcare in police custody in the Netherlands. The indicators are intended for use by care providers in police custody and oversight bodies to monitor quality, compare performance across regions, and identify opportunities for improvement. They can also inform policy discussions on workforce allocation, interagency collaboration, and detainee safety. Establishing a regular review process for updating the indicator set as evidence and systems evolve will be essential to maintain its relevance and impact.

## Supplementary Information

Supplementary Material 1. Long list of quality indicators for police custody healthcare.

## Data Availability

The data supporting the conclusions of this article are included within the article and its supplementary file.
