# Supplementary material for: Measuring healthcare quality in police custody: the development of quality indicators through expert consultations in the Netherlands
Source: Health Justice. 2026 Aug 5;14:34. doi: 10.1186/s40352-026-00415-2 (PMC13445773; doi:10.1186/s40352-026-00415-2)
Supplement: Supplementary file 1 [file 40352_2026_415_MOESM1_ESM.pdf]

Supplementary Table I: Long list of quality indicators for healthcare in police custody

#	Domain	Subdomain	Indicator
**STRUCTURE INDICATORS**			
1	Legal framework	Access rights to medical records	Is there nationwide access to information about previous consultations and the medical history of the detainees?
2	Legal framework	Access rights to medical records	Are there between the healthcare provider organization and mental health services in the region established agreements on how relevant information from a record can be obtained?
3	Legal framework	Access rights to medical records	Are there between the healthcare provider organization and addiction care organizations in the region established agreements on how relevant information from a record can be obtained (e.g., medication dosage)?
4	Legal framework	Access rights to medical records	Does the healthcare provider organization have access to the National Exchange Point?
5	Legal framework	Access rights to medical records	Is there clarity for the healthcare providers about when medical information may be shared between healthcare providers and who may access what information (e.g., established agreements, protocols or guidelines)?
6	Legal framework	Medical confidentiality	Does the healthcare provider organization have protocols/work instructions in place on how to deal with the tension between medical confidentiality and safe healthcare provision when sharing medical data?
7	Legal framework	Medical confidentiality	In cases where the police distribute the medication to detainees: is there a work process in place whereby medication can be delivered to the detainee in accordance with medical confidentiality (e.g., in packages bearing only the detainee's name and time of administration)?
8	Scope and nature of the care demand	Availability of information about the care demand	Is information on diagnoses and treatments/procedures performed by healthcare providers systematically recorded? At least:<br>- Distinction between somatic and mental health complaints / disorders;<br>- Structured classification of health complaints, symptoms and disorders (e.g., use of International Classification of Primary Care);<br>- Distinction between types of procedures.
9	(Healthcare) staff	Capacity and skill-mix	Availability of the number and type of staff per day in the police custody facility, broken down by day and night times
10	(Healthcare) staff	Capacity and skill-mix	Availability of the number and type of healthcare staff (specialist physician, physician, nurse) per police cell in the service area of the healthcare provider organization, broken down by day and night times
11	(Healthcare) staff	Capacity and skill-mix	Number of police custody officers certified as Emergency Response Officer (ERO), as a percentage of the total number of police custody officers
12	(Healthcare) staff	Capacity and skill-mix	Number of police custody officers holding a First Aid certificate, as a percentage of the total number of police custody officers
13	(Healthcare) staff	Education and training	Number of police custody officers who have completed the training for police custody officer, as a percentage of the total number of police custody officers

14	(Healthcare) staff	Education and training	Does the education/training for the police cover the general recognition, detection and description of unusual or urgent medical situations?
15	(Healthcare) staff	Education and training	Does the education/training for the police cover the recognition of an Excited Delerium Syndrome?
16	(Healthcare) staff	Education and training	Does the education/training for the healthcare providers cover the recognition of an Excited Delerium Syndrome?
17	(Healthcare) staff	Education and training	Does the police have an organization-wide policy on training and education related to knowledge of specific populations, their health needs, associated health risks, and police conduct toward them?
18	(Healthcare) staff	Education and training	Does the healthcare provider organization have an organization-wide policy on training and education related to knowledge of specific populations, their health needs, associated health risks, and police conduct toward them?
19	(Healthcare) staff	Education and training	Does the training/education of the police cover the appropriate interaction with detainees with a (mild) intellectual disability?
20	(Healthcare) staff	Education and training	Does the training/education of the healthcare providers cover the appropriate interaction with detainees with a (mild) intellectual disability?
21	(Healthcare) staff	Education and training	Number of healthcare providers who completed education or training specifically focused on healthcare provision in police custody, as a percentage of the total number of healthcare providers
22	(Healthcare) staff	Education and training	In cases where the police carry out the medication rounds: have those who carry out the medication rounds received specific training for this?
23	(Healthcare) staff	Education and training	If applicable: what is the job profile/training/knowledge of control room staff handling the calls from the police to be referred to the healthcare providers?
24	Quality assurance	Standardization of work processes	Do the police have a guideline on when someone is taken directly to the mental health crisis assessment location (without first being held at a police custody facility)?
25	Quality assurance	Standardization of work processes	Do the police have a standardized questionnaire about detainees' medical care needs that the police custody officers can use at intake to make an initial assessment of the health situation and health risks of detainees?
26	Quality assurance	Standardization of work processes	Do the police have a protocol or work instruction specifying which staff members (in terms of role and competencies) are responsible for administering the questionnaire on medical care needs?
27	Quality assurance	Standardization of work processes	Has the standardized questionnaire on detainees' medical care needs, that the police can use at intake, been developed in consultation with healthcare providers?
28	Quality assurance	Standardization of work processes	Do the healthcare provider organizations have a (standardized) triage method in use?
29	Quality assurance	Standardization of work processes	Do the healthcare provider organizations have a work procedure in place whereby in written form (on paper or digitally) an explanation and instructions are left regarding monitoring and treatment at the police custody facility?

30	Quality assurance	Standardization of work processes	Do the healthcare provider organizations have a work procedure in place whereby in written form (on paper or digitally) an explanation and instructions are left regarding medication at the police custody facility?
31	Quality assurance	Standardization of work processes	Are there work instructions regarding which medication and dosage may or may not be given to the detainee upon discharge?
32	Quality assurance	Standardization of work processes	Is there a protocol on how to handle suicidal behavior?
33	Quality assurance	Standardization of work processes	Is there a protocol on how to handle detainee who refuse treatment or medication?
34	Quality assurance	Registration systems	Do the police use a registration system in which monitoring (health-related checks of detainees) is recorded?
35	Quality assurance	Registration systems	Do the healthcare organizations have a registration system (electronic health record) in place for medical care in which at least can be registered: relevant medical history, diagnosis and treatment, advice, medication and allergies?
36	Quality assurance	Registration systems	In cases where the police distribute the medication: do they have a registration system in place to record the medication rounds?
37	Quality assurance	Registration systems	In cases where the healthcare providers distribute the medication: do they have a registration system in place to record the medication rounds?
38	Quality assurance	Registration systems	In cases where sometimes the police and sometimes the healthcare provider distribute the medication: is there a system in place through which information can be exchanged about the medication rounds?
39	Quality assurance	Responsibilities	Do the healthcare provider organizations have (formal) agreements in place outlining the roles and responsibilities of physician specialists, junior doctors, and nurses?
40	Quality assurance	Responsibilities	Is there a healthcare professional designated as the lead responsible for medication storage?
41	Quality assurance	Agreements between police and healthcare provider	Are there agreements between the police and the healthcare provider organization about when a healthcare provider should be contacted?
42	Quality assurance	Agreements between police and healthcare provider	Do the police have work instructions available and accessible, clearly outlining: when, how, and which healthcare provider should be contacted (e.g., phone numbers including emergency number, if applicable clarification when a physician and when a nurse should be contacted)?
43	Quality assurance	Agreements between police and healthcare provider	Do the police have procedures in place defining who is responsible for contacting a healthcare provider?
44	Quality assurance	Agreements between police and healthcare provider	Are there between the police and the healthcare provider organization established agreements about the scope and setup of the collaboration and the involvement in the care for detainees?

45	Quality assurance	Agreements between police and healthcare provider	Are there established agreements between the police, the healthcare provider organization, and pharmacies about the delivery and/or pickup of medication, both during and outside pharmacy office hours?
46	Quality assurance	Agreements between police and healthcare provider	In cases where the police distribute the medication to detainees: are there established agreements between the police and the healthcare provider organization about how and when this distribution of medication should be executed?
47	Quality assurance	Agreements between police and healthcare provider	Are there between the police, the healthcare provider organization, and the mental health crisis service established agreements about the scope and setup of the collaboration and the involvement of each party in the care provision for individuals with confused or challenging behavior held in police custody?
48	Quality assurance	Agreements between police and healthcare provider	Is there systematic consultation between the police and the mental health crisis service about the collaboration in the care provision for individuals with confused or challenging behavior held in police custody?
49	Quality assurance	Agreements between healthcare providers	Do the healthcare provider organizations have established opportunities for peer consultation, both routinely and in acute situations?
50	Quality assurance	Agreements between healthcare providers	Are there agreements between the healthcare provider organization and the mental health crisis service about when the mental health crisis service is contacted for an assessment?
51	Quality assurance	Agreements between healthcare providers	Are there agreements between the healthcare provider organization and the mental health crisis service regarding who takes the lead when both parties are involved?
52	Quality assurance	Agreements between healthcare providers	Are there agreements between the healthcare provider organization and chain partners (such as addiction care services) regarding the possibility of interprofessional consultation?
53	Quality assurance	Handling of complaints and incidents	Do the police have a procedure in place for (former-)detainees to report complaints about the care and medical care within the police custody facility?
54	Quality assurance	Handling of complaints and incidents	Do the police have a procedure in place for healthcare providers to report complaints about the care services within the police custody facility?
55	Quality assurance	Handling of complaints and incidents	Do the healthcare provider organizations have a procedure in place for (former-)detainees to report complaints about the medical care within the police custody facility?
56	Quality assurance	Handling of complaints and incidents	Do the healthcare provider organizations have a procedure in place for police to report complaints about the medical care within the police custody facility?
57	Quality assurance	System for quality assurance and quality improvement	Do the police organize at least once a year peer review meetings, during which attention is also paid to (medical) care provision?

58	Quality assurance	System for quality assurance and quality improvement	Do the healthcare provider organizations organize at least once a year peer review meetings, during which attention is paid to medical care issues that have arisen?
59	Infrastructure and (medical) resources	Police cell provisions	Do the police custody facility have facilities available for detainees with physical disabilities or wheelchair users?
60	Infrastructure and (medical) resources	Police cell provisions	Is there a clock in the overnight cells so that detainees can see the time?
61	Infrastructure and (medical) resources	Police cell provisions	Do the police custody facilities have cells in available in which detainees can be monitored while awaiting the arrival of a healthcare provider?
62	Infrastructure and (medical) resources	(Medical) equipment	Do the police custody facilities have blood glucose meters available for detainees with insulin-dependent diabetes?
63	Infrastructure and (medical) resources	(Medical) equipment	Is there an established structure in place that available medical equipment and materials (e.g., blood glucose monitors) are periodically maintained, verified, or calibrated?
64	Infrastructure and (medical) resources	Medication	Are there a lockable medicine cabinet available in the police custody facilities?
65	Infrastructure and (medical) resources	Medication	Are there an emergency or working stock of medications available at the custody facilities or with the healthcare provider, tailored to the care needs of detainees (particularly for addiction care)? (e.g., methadone, diazepam, oxazepam, painkillers (paracetamol, ibuprofen), tetanus vaccine, insulin pens, and pregabalin?
66	Infrastructure and (medical) resources	Medication	Are there at the police custody facilities products with fast carbohydrates available for detainees with diabetes?
67	Infrastructure and (medical) resources	Interpreter	Is it (always) possible to call on the help of an interpreter?
68	Infrastructure and (medical) resources	Clinical diet	Are there formal arrangements in place to ensure that food and drinks are provided in accordance with medical and philosophical or religious requirements?
69	Funding	Funding of healthcare provision	Is there insight into the budgetary framework within which the police custody healthcare is provided?
70	Funding	Funding of treatment	Are there arrangements for the medical care provision (treatment, medication, etc.) of uninsured detainees?
**PROCESS INDICATORS**			
71	Triage and access to healthcare	Triage	Is the inquiry on medical care needs by the police (i.e., questionnaire about detainee's medical care needs) registered?
72	Triage and access to healthcare	Triage	Number of detainees for whom the standardized questionnaire about medical care needs has been fully completed by the police at intake, as a percentage of the total number of detainees

73	Triage and access to healthcare	Triage	Time between arrest by the police and intake at the police station where medical care needs are inquired
74	Triage and access to healthcare	Triage	Time between arrival at the police station and intake where medical care needs are inquired
75	Triage and access to healthcare	Triage	Number of times the action resulting from the questionnaire about the detainee’s medical care needs was recorded by the police, as a percentage of the total number of detainees for whom the questionnaire was fully completed
76	Triage and access to healthcare	Triage	Number of times it was recorded who needed to be contacted and who was contacted (e.g., physician, nurse, mental health crisis service), as a percentage of the total number of detainees who required a healthcare provider to be contacted
77	Triage and access to healthcare	Timely contact with healthcare provider	Time between the identification of a healthcare need by the police and the first moment of contact with the healthcare provider by the police
78	Triage and access to healthcare	Timely contact with healthcare provider	Number of detainees for whom a physician was contacted in cases of intoxication (e.g., alcohol, drugs, medication), as a percentage of the total number of detainees with an intoxication
79	Triage and access to healthcare	Timely contact with healthcare provider	In cases where a healthcare provider was contacted in relation to intoxications (e.g., alcohol, drugs, medication): time between the police identifying the need to contact a healthcare provider and the actual moment of contact between the healthcare provider and the police
80	Triage and access to healthcare	Timely contact with healthcare provider	Number of detainees who had a face-to-face consultation with a healthcare provider for drug-related issues in cases where: there was no medical record available, there was no access to the medical record, or the case involved an acute drug intoxication, as a percentage of the total number of detainees with drug-related issues
81	Triage and access to healthcare	Timely contact with healthcare provider	In cases where a healthcare provider was contacted due to acute drug intoxication and no medical record was available: time between the police identifying the need to contact a healthcare provider and the actual moment the healthcare provider visits the detainee
82	Triage and access to healthcare	Timely contact with healthcare provider	Number of detainees from whom a healthcare provider was contacted because they indicated having insulin-dependent type 1 diabetes, as a percentage of the total number of detainees with insulin-dependent type 1 diabetes
83	Triage and access to healthcare	Timely contact with healthcare provider	In cases where a healthcare provider was contacted for insulin-dependent diabetes: time between the police identifying the need to contact a healthcare provider and the actual moment of contact between the healthcare provider and the police
84	Triage and access to healthcare	Timely contact with healthcare provider	Number of detainees for whom complaints of chest pain led to an assessment by a healthcare provider (either in police custody or by ambulance personnel), as a percentage of the total number of detainees with chest pain complaints

85	Triage and access to healthcare	Timely contact with healthcare provider	In cases where a healthcare provider was contacted regarding chest pain: time between the police identifying the need to contact a healthcare provider and the actual moment of contact between the healthcare provider and the police
86	Triage and access to healthcare	Timely contact with healthcare provider	Number of detainees for whom new/acute neurological deficits (suspected stroke) led to an assessment by a healthcare provider (either in police custody or by ambulance personnel), as a percentage of the total number of detainees with neurological deficits
87	Triage and access to healthcare	Timely contact with healthcare provider	In cases where a healthcare provider was contacted for new/acute neurological deficits (suspected stroke): time between the police identifying the need to contact a healthcare provider and the actual moment of contact between the police and the healthcare provider
88	Triage and access to healthcare	Timely contact with healthcare provider	Number of detainees who indicated having epilepsy and for whom the police contacted a healthcare provider, as a percentage of the total number of detainees who indicated having epilepsy
89	Triage and access to healthcare	Timely contact with healthcare provider	In cases where a healthcare provider was contacted for epilepsy: time between the identification of this condition by the police and their contact with the healthcare provider
90	Triage and access to healthcare	Timely contact with healthcare provider	Number of detainees with accident-related injuries for whom the police contacted a physician, as a percentage of all detainees with such injuries
91	Triage and access to healthcare	Timely contact with healthcare provider	In cases where a healthcare provider was contacted for accident-related injury: time between the police identifying the injury and their contact with the healthcare provider
92	Triage and access to healthcare	Timely contact with healthcare provider	Number of detainees referred to a hospital following an accident at a speed exceeding 40-50 km/h, as a percentage of the total number of detainees involved in accidents at that speed
93	Triage and access to healthcare	Timely contact with healthcare provider	In cases where a healthcare provider was contacted after an accident at speeds exceeding 40-50 km/h: time between the identification of this by the police and their contact with a healthcare provider
94	Triage and access to healthcare	Timely contact with healthcare provider	Number of detainees who were seen by a healthcare provider in cases where a Taser was used, as a percentage of the total number of detainees for whom a Taser was used
95	Triage and access to healthcare	Timely contact with healthcare provider	In cases where a healthcare provider was contacted after the use of a Taser: time between the identification of this by the police and their contact with a healthcare provider
96	Triage and access to healthcare	Timely contact with healthcare provider	Number of detainees for whom a forensic physician was contacted in cases where restraint devices were used, as a percentage of the total number of detainees for whom restraining devices were used
97	Triage and access to healthcare	Timely contact with healthcare provider	In cases where a healthcare provider was contacted after the use of restraint devices: time between the application of the restraint and the contact with a healthcare provider

98	Triage and access to healthcare	Timely contact with healthcare provider	In cases where a healthcare provider was contacted regarding excited delirium syndrome symptoms: time between the police's recognition and the contact with a healthcare provider
99	Triage and access to healthcare	Timely contact with healthcare provider	In cases where a detainee was carried inside by the police in a lying/horizontal position: number of detainees who were placed in a holding cell with camera surveillance until (s)he had been seen by a physician, as percentage of the total detainees who were carried inside in a lying/horizontal position
100	Triage and access to healthcare	Timely contact with healthcare provider	In cases where a healthcare provider was contacted regarding a detainee who was carried inside in a lying/horizontal position: time between the police's recognition and the contact with a healthcare provider
101	Triage and access to healthcare	Timely access to medication	Number of detainees who were given medication on time as prescribed, as a percentage of all detainees who needed medication
102	Triage and access to healthcare	Timely access to medication	Number of times when the required medication was not in stock and therefore did not reach the detainee on time, as a percentage of the total number of medication prescriptions
103	Triage and access to healthcare	Timely access to medical assessment	Time between contacting a healthcare provider and the consultation with a healthcare provider:<br>- by urgency level<br>- stratified by face-to-face (physical) and telephone consultations
104	Triage and access to healthcare	Timely access to medical assessment	Time between (recommendation to) contact the mental health crisis service and arrival of/at mental health crisis service, broken down by urgency level or type of help request
105	Triage and access to healthcare	Timely access to medical assessment	Number of detainees who received an in-person assessment by a mental health crisis service provider within 2 hours after the initial phone contact, as a percentage of the total number of detainees with a high urgency level for mental health assessment
106	Triage and access to healthcare	Timely referral	Number of detainees taken/sent to the hospital after police arrest, as a percentage of the total number of detainees
107	Triage and access to healthcare	Timely referral	Time between the decision that a detainee had to be taken to the hospital and the start of the transport to the hospital
108	Detection of care needs	Detection of care needs of detainees' care demand	Number of detainees for whom a healthcare provider was contacted, as a percentage of the total number of detainees
109	Detection of care needs	Detection of care needs of detainees' care demand	Number of detainees who received a face-to-face consultation, as a percentage of the total number of detainees
110	Detection of care needs	Detection of care needs of detainees' care demand	Number of consultations in which an interpreter was involved, as a percentage of the total number of consultations

111	Detection of care needs	Detection of care needs of detainees’ care demand	Number of consultations in which an interpreter was involved, as a percentage of the total number of detainees with health needs who were not proficient in Dutch or English
112	Detection of care needs	Detection of care needs of detainees’ care demand	Number of occasions on which a healthcare provider was contacted concerning detainees who refused medical care despite indications that this was required, as a percentage of the total number of detainees who refused care despite indications that is was necessary
113	Detection of care needs	Detection of care needs of detainees’ care demand	Number of detainees who were assessed by a healthcare provider regarding excited delirium symptoms, as a percentage of the total number of detainees
114	Detection of care needs	Monitoring	Number of instances in which detainees were monitored at two-hour intervals (while in their cells), presented as a percentage of the total number of detainees
115	Detection of care needs	Monitoring	Number of instances in which detainees awaiting a physician were monitored at least every 15 minutes (while in their cells), as a percentage of the total number of detainees who waited for a physician
116	Detection of care needs	Monitoring	Number of instances where the healthcare providers’ instructions about monitoring were followed, as a percentage of the total number of detainees about whom a healthcare provider gave instructions about monitoring
117	Detection of care needs	Monitoring	Number of cases in which a medical consultation with a physician took place for stays exceeding 24 hours in an observation cell, as a percentage of the total number of detainees who stayed longer than 24 hours in an observation cell
118	Continuity of healthcare information	(Timely) availability of medical information	In the event of hospital admission: number of times relevant information about the hospital admission (at minimum: advice and medication use) was transferred to the healthcare provider, as a percentage of the total number of hospital admissions between arrest and stay in police custody facility
119	Continuity of healthcare information	(Timely) availability of medical information	In cases where the detainee has given consent: number of detainees for whom an up-to-date medication list was obtained within 24 hours, as a percentage of the total number of detainees who have given consent
120	Continuity of healthcare information	(Timely) availability of medical information	In cases where a detainee has given consent: number of detainees for whom information from the general practitioner was obtained within 24 hours, as a percentage of the total number of detainees who have given consent to request information from the general practitioner
121	Continuity of healthcare information	(Timely) availability of medical information	Number of detainees whose identity was known, registered, and verified, as a percentage of the total number of detainees

122	Continuity of healthcare information	Communication	In cases where the monitoring/treatment advice deviated: number of times the healthcare provider left a written explanation and instruction (on paper or digitally), as a percentage of the total number of cases where the monitoring/treatment advice deviated
123	Continuity of healthcare information	Communication	Number of times the health care records kept by the healthcare provider contains at least detainee's: date of birth, sex, and International Classification of Primary Care code, as a percentage of the total number of reviewed records
124	Continuity of healthcare information	Communication	Number of times the police custody officers indicated that they understood the healthcare provider's advice, as a percentage of the total number of times the police custody officers where asked whether they understood the advice
125	Coordination of care	Coordination of transition	In case of transfer to a custodial facility (e.g., pre-trial detention facility): number of times medical information had been shared with the healthcare provider in the custodial facility, as a percentage of the total number of detainees who were transferred from police custody to a custodial facility
126	Coordination of care	Coordination of transition	In case of transfer to a custodial facility (e.g., pre-trial detention facility): number of times information from the health record had been shared with the healthcare provider in the custodial facility, as a percentage of the total number of detainees who were transferred from police custody to a custodial facility and received healthcare in police custody
127	Coordination of care	Coordination of transition	In case of transfer to a custodial facility (e.g., pre-trial detention facility): time between the request for a medical file from the pre-trial detention facility and the sending of the medical file by the police custody healthcare provider
128	Coordination of care	Coordination of transition	In case of transfer to a custodial facility (e.g., pre-trial detention facility): number of times a medical file was transferred to the pre-trial detention facility before the detainee arrived at the pre-trial detention facility, as a percentage of the total number of detainees who were sent to a custodial facility and received medical care in police custody
129	Coordination of care	Coordination of transition	In case of transfer to a custodial facility (e.g., pre-trial detention facility): number of times the medication administration list was sent or handed to the detainee, as a percentage of the total number of detainees who needed medication and were sent to a custodial facility
130	Coordination of care	Coordination of transition	In case of pre-trial detention: Number of times medication for the next 24 hours was given when transferring to the pre-trial detention facility, as a percentage of the total number of detainees who needed medication and were sent to a custodial facility
131	Coordination of care	Coordination of transition	In case of release from police custody: number of times information was sent to the detainee's general practitioner, as a percentage of the total number of detainees who received medical care in police custody

132	Coordination of care	Coordination of transition	In case of release and if the detainee is known to a healthcare institution: number of times the relevant medical reports were provided or sent during transfer to a chain partner other than the custodial institutions, as a percentage of the total number of detainees who were known to a healthcare institution, received care, and were released
133	Coordination of care	Coordination of transition	In case of release and if the detainee is known to a healthcare institution: number of times the medication administration list was sent or handed to the detainee, as a percentage of the total number of detainees who were known to a healthcare institution, needed medication, and were released
134	Coordination of care	Coordination of transition	Number of transfers between cell complexes during the detention period by the police (e.g., between the police station and custody facility for overnight stays)
135	Healthcare provision (general)	Identification and treatment of urgent health problems	Insights into activities related to medical care:<br>- Frequently registered ICPC codes<br>- Percentage of face-to-face and telephone consultations<br>- Type(s) of involved healthcare providers (e.g., physician, nurse).<br>- Percentage of detainees referred to the hospital<br>- Percentage of detainees who received a fitness for custody assessment, and description of the outcome of the assessment
136	Healthcare provision (general)	Identification and treatment of urgent health problems	Number of times medical confidentiality is mentioned, as a percentage of the total number of detainees who have spoken with a healthcare provider (in person) (e.g., at the interface between police custody care and forensic medical expertise)
137	Healthcare provision (general)	Prescription and dispension of medication	In case medication was prescribed: number of detainees for whom medication was verified (e.g., with attending physician or through the National Exchange Point), as a percentage of the total number of detainees who were prescribed medication
138	Healthcare provision (general)	Prescription and dispension of medication	In case medication was prescribed: number of times it was indicated that the detainee had given consent for consultation of the National Exchange Point, as a percentage of the total number of detainees who were prescribed medication
139	Healthcare provision (general)	Prescription and dispension of medication	In case medication was prescribed and the detainee provided consent for consultation of the National Exchange Point: Number of times medication was verified through the National Exchange Point, as a percentage of the total number of detainees who gave consent for consultation of the National Exchange Point
140	Healthcare provision (general)	Prescription and dispension of medication	In case medication was required and brought by the detainee or someone else: number of times a check was carried out to ensure the medication was not expired, as a percentage of the number of detainees who had their own medication available

141	Healthcare provision (general)	Prescription and dispension of medication	In case of detention exceeding 24 hours: number of cases in which at least the following information was known: current medication use (including "as needed" medication), contraindications, drug allergies, and medication history for the past three months), as a percentage of the total number of detainees held for at least 24 hours who reported using medication
142	Healthcare provision (general)	Prescription and dispension of medication	In cases where medication was prescribed: number of times the medication administration instruction was registered in the health record, as a percentage of the total number of detainees for whom medication was prescribed
143	Healthcare provision (general)	Prescription and dispension of medication	In case medication was prescribed: number of times the administration list was complete and up to date, as a percentage of the total number of detainees who were prescribed medication
144	Healthcare provision (general)	Prescription and dispension of medication	In case medication was prescribed: number of times the medicines were present in labeled packaging, as a percentage of the total number of detainees who were prescribed medication
145	Healthcare provision (general)	Prescription and dispension of medication	In case medication remained after the release of a detainee: are these destroyed?
146	Healthcare provision (general)	Complaints handling	Number of times complaints were handled by the police in accordance with the established procedure, as a percentage of the total number of complaints
147	Healthcare provision (general)	Complaints handling	Number of times complaints were handled by the healthcare provider organization in accordance with the established procedure, as a percentage of the total number of complaints
148	Healthcare provision (common needs)	Drug withdrawal	Number of cases in which GHB dependence was assessed shortly after being placed in a police cell, as a percentage of the total number of detainees with an intoxication
149	Healthcare provision (common needs)	Drug withdrawal	Number of cases in which detainees with acute intoxication (e.g., GHB) were seen by a physician, as a percentage of the total number of detainees with an acute intoxication
150	Healthcare provision (common needs)	Drug withdrawal	Number of cases in which contact was made with the mental health crisis service for acute psychotic reactions (e.g., due to cannabis), paranoid delusions, and/or hallucinations, as a percentage of the total number of detainees experiencing acute psychotic reactions, paranoid delusions, and/or hallucinations
151	Healthcare provision (common needs)	Drug withdrawal	Number of cases in which opioid substitution therapy was initiated within 24 hours of detention (or earlier), as a percentage of the total number of detainees

152	Healthcare provision (common needs)	Drug withdrawal	Number of cases in which documentation of physical consultations for intoxications in the (electronic) medical record included at least the following items in understandable language:<br>- Reason for detention, insofar as medically relevant<br>- Start time of detention<br>- Time of examination<br>- (suspected) substances used<br>- Behavior and level of consciousness<br>- Breathing and circulation<br>- Pupil size and reactions<br>- Motor skills / spontaneous movements<br>- Management, monitoring, wake-up advice, and instructions for custodial staff<br>as a percentage of the total number of randomly reviewed medical records
153	Healthcare provision (common needs)	Alcohol withdrawal	Number of cases in which detainees with a (suspected) intoxication were placed in an observation cell, as a percentage of the total number of detainees with a (suspected) intoxication
154	Healthcare provision (common needs)	Mental/psychiatric health problems	Number of cases in which aggressive individuals or persons exhibiting confused or challenging behavior, who were restrained with significant force by the police, were assessed by a physician within 15-30 minutes after arrest, as a percentage of the total number of detained aggressive individuals or persons exhibiting confused or challenging behavior who were restrained with significant force by the police
155	Healthcare provision (common needs)	Mental/psychiatric health problems	Number of cases in which aggressive individuals or persons exhibiting confused or challenging behavior, who were restrained with significant force by the police, were monitored/observed until the physician arrived, as a percentage of the total number of detained aggressive individuals or persons exhibiting confused or challenging behavior who were restrained with significant force by the police
156	Healthcare provision (common needs)	Mental/psychiatric health problems	Number of cases detainees with symptoms of Excessive Delirium Syndrome (EDS) were transferred to the hospital, as a percentage of the total number of detainees
157	Healthcare provision (common needs)	Self-harm/suicidal behavior	Number of cases a physician or psychiatrist was consulted when it was reasonably assumed that a detainee would attempt suicide, as a percentage of the total number of detainees
158	Healthcare provision (common needs)	Intellectual disability	Number of cases in which a physician was contacted when there was an impression that a detainee had an intellectual disability, as a percentage of the total number of detainees
159	Healthcare provision (common needs)	Minors	Number of cases in which a healthcare provider was contacted for minors, as a percentage of the total number of detained minors

160	Healthcare provision (common needs)	Minors	Number of cases in which a physician granted permission for overnight stay in a police cell, as a percentage of the total number of overnight stays by minors
**OUTCOME INDICATORS**			
161	Effectiveness and health	Mortality	Number of deaths in police custody, broken down by region, age, and (medical) characteristics
162	Effectiveness and health	Mortality	Description of recorded deaths in police custody
163	Effectiveness and health	Mortality	Assessment of the preventability of (potentially) healthcare-related deaths in police custody
164	Effectiveness and health	Incidents	Number of registered healthcare-related incidents, as a percentage of the total number of detainees (incident defined as: an unintended or unexpected event that relates to the quality of care, and which has led, could have led, or might lead to harm to the client)
165	Effectiveness and health	Incidents	Description of recorded healthcare-related incidents (incident defined as: an unintended or unexpected event that relates to the quality of care, and which has led, could have led, or might lead to harm to the client)
166	Effectiveness and health	Incidents	Assessment of preventability of (potentially) healthcare-related incidents
167	Effectiveness and health	Incidents	Have patient safety incident reports been followed up as described in the protocol?
168	Effectiveness and health	Complications	Number of registered healthcare-related complications
169	Effectiveness and health	Complications	Description of recorded healthcare-related medical complications
170	Satisfaction	Satisfaction and complaints of detainees	Number of care-related complaints of detainees about the police, as a percentage of the total number of detainees
171	Satisfaction	Satisfaction and complaints of detainees	Description of care-related complaints of detainees about the police
172	Satisfaction	Satisfaction and complaints of detainees	Number of healthcare-related complaints of detainees about the healthcare providers, as a percentage of the total number of detainees
173	Satisfaction	Satisfaction and complaints of detainees	Description of healthcare-related complaints of detainees about the healthcare providers, as a percentage of the total number of detainees
174	Satisfaction	Satisfaction and complaints of detainees	Satisfaction of detainees with the conduct of the police
175	Satisfaction	Satisfaction and complaints of detainees	Satisfaction of detainees with the conduct of the healthcare providers
176	Satisfaction	Satisfaction and complaints of healthcare providers	Number of times healthcare providers felt they were contacted in a timely manner, as a percentage of the total number of times the question was asked

177	Satisfaction	Satisfaction and complaints of healthcare providers	Satisfaction of healthcare providers with the measures available to safeguard medical confidentiality
178	Satisfaction	Satisfaction and complaints of healthcare providers	Satisfaction of healthcare providers about the (quality of the) guidelines and protocols
179	Satisfaction	Satisfaction and complaints of healthcare providers	Satisfaction of healthcare providers about administrative burden
180	Satisfaction	Satisfaction and complaints of healthcare providers	Satisfaction of the police custody healthcare organization about agreements and actual practice about when they are contacted by the police
181	Satisfaction	Satisfaction and complaints of the police	Satisfaction of the police about administrative burden regarding care
182	Satisfaction	Satisfaction and complaints of the police	Satisfaction of the police about the provided healthcare by the healthcare providers (other than response times)
183	Satisfaction	Satisfaction and complaints of the police	Satisfaction of the police about the response times of the healthcare providers
184	Satisfaction	Satisfaction and complaints of the police	Satisfaction of the police about the agreements for the delivery and/or pickup of medication during pharmacy office hours
185	Satisfaction	Satisfaction and complaints of the police	Satisfaction of the police about the agreements for the delivery and/or pickup of medication outside pharmacy office hours
